# Could it be hereditary angioedema?—Perspectives from different medical specialties

**DOI:** 10.1002/clt2.12297

**Published:** 2023-09-19

**Authors:** Markus Magerl, Anna Sala‐Cunill, Christina Weber‐Chrysochoou, Susanne Trainotti, Ilaria Mormile, Giuseppe Spadaro

**Affiliations:** ^1^ Institute of Allergology IFA Charité – Universitätsmedizin Berlin Berlin Germany; ^2^ Fraunhofer Institute for Translational Medicine and Pharmacology ITMP Allergology and Immunology Berlin Germany; ^3^ Allergy Section Internal Medicine Department Hospital Universitari Vall d’Hebron Barcelona Spain; ^4^ Allergy Unit Dermatology Department University Hospital of Zurich Zurich Switzerland; ^5^ Department of Otorhinolaryngology Klinikum rechts der Isar Technical University of Munich Munich Germany; ^6^ Department of Translational Medical Sciences and Center for Basic and Clinical Immunology Research (CISI) University of Naples Federico II Naples Italy

**Keywords:** bradykinin, C1‐esterase inhibitor, differential diagnosis, hereditary angioedema, rare disease

## Abstract

Hereditary angioedema (HAE) is a rare autosomal dominant disease, with patients often suffering with associated symptoms for many years before receiving a correct diagnosis. The symptoms greatly impact a patient's quality of life (QoL) and include excruciating abdominal pain and angioedema of the skin and submucosa. Angioedema of the larynx represents a significant mortality risk in undiagnosed patients, and a large proportion of patients with HAE receive incorrect diagnoses and undergo unnecessary surgery. HAE‐specific treatments can control and prevent acute life‐threatening episodes, in addition to improving QoL, emphasizing the value of early diagnosis for patients. Diagnostic delay may be due to a lack of HAE awareness by healthcare professionals and the similarity of HAE symptoms with those of more common conditions, complicating differential diagnosis. The multifaceted nature of the condition may result in visits to one of many different medical settings, for example: the Emergency Room, pediatrics, general practice, otolaryngology, gastroenterology, and dermatology. Therefore, it is crucial that physicians in multiple healthcare specialties are aware of the disease to ensure that patients with HAE receive a timely diagnosis. Using patient cases from various medical specialties, this review highlights the necessity for cross‐specialty awareness of HAE and outlines the essential information for the various healthcare professionals that may encounter a patient with HAE symptoms, in order to effectively treat and/or diagnose HAE.

## 1 INTRODUCTION

*Patient case—The importance of early diagnosis*

*A woman, aged 53, attended a hereditary angioedema (HAE) specialist center in 2008 after reading an article in the lay press on the disease. Her symptoms first appeared when she was 7 years old. Since then, she has experienced skin swelling 2–3 times a month and severe abdominal pain twice a year, on average. The abdominal pain would last for up to 7 days. As a child, the patient's symptoms were attributed to insect bites, allergy, and lactose intolerance, and, unable to confirm a proper diagnosis, was given a diagnosis of simply being ‘sensitive’. In 1967, aged 12, following an attack of extreme abdominal pain, the patient was diagnosed with appendicitis, and underwent an appendectomy. When symptoms continued after the appendectomy, the patient felt that her friends and family did not believe her. At age 38, in 1993, she underwent a hysterectomy, in a further attempt to relieve the pain. This also failed to prevent the symptoms she was experiencing; the patient was prescribed painkillers and was resigned to the fact that her condition was a mystery. After reading an article in the lay press about HAE, she visited an HAE specialist center, noting that she experiences similar symptoms to those described. When questioned about her family history, she noted that her daughter has similar complaints, as did her father. Her consultant at the HAE specialist center ordered tests for C1 inhibitor (C1‐INH) function and concentration, and C4. Her results confirmed a diagnosis of HAE type II (C1‐INH concentration: 300 mg/L [normal range 180–320 mg/L]; C1‐INH function: 11% [normal range 70%–130%]; C4: 40 mg/L [normal range 100–400 mg/L]; missense mutation in exon 8), after which her disease was effectively managed with plasma‐derived C1‐INH replacement therapy—over 40 years after the onset of symptoms. During this time, the patient was consulted by pediatricians, allergists, dermatologists, gastroenterologists, otolaryngologists, gynecologists, Emergency Room (ER) physicians and surgeons, highlighting the importance of HAE awareness across multiple medical specialties*.

**FIGURE 1:**
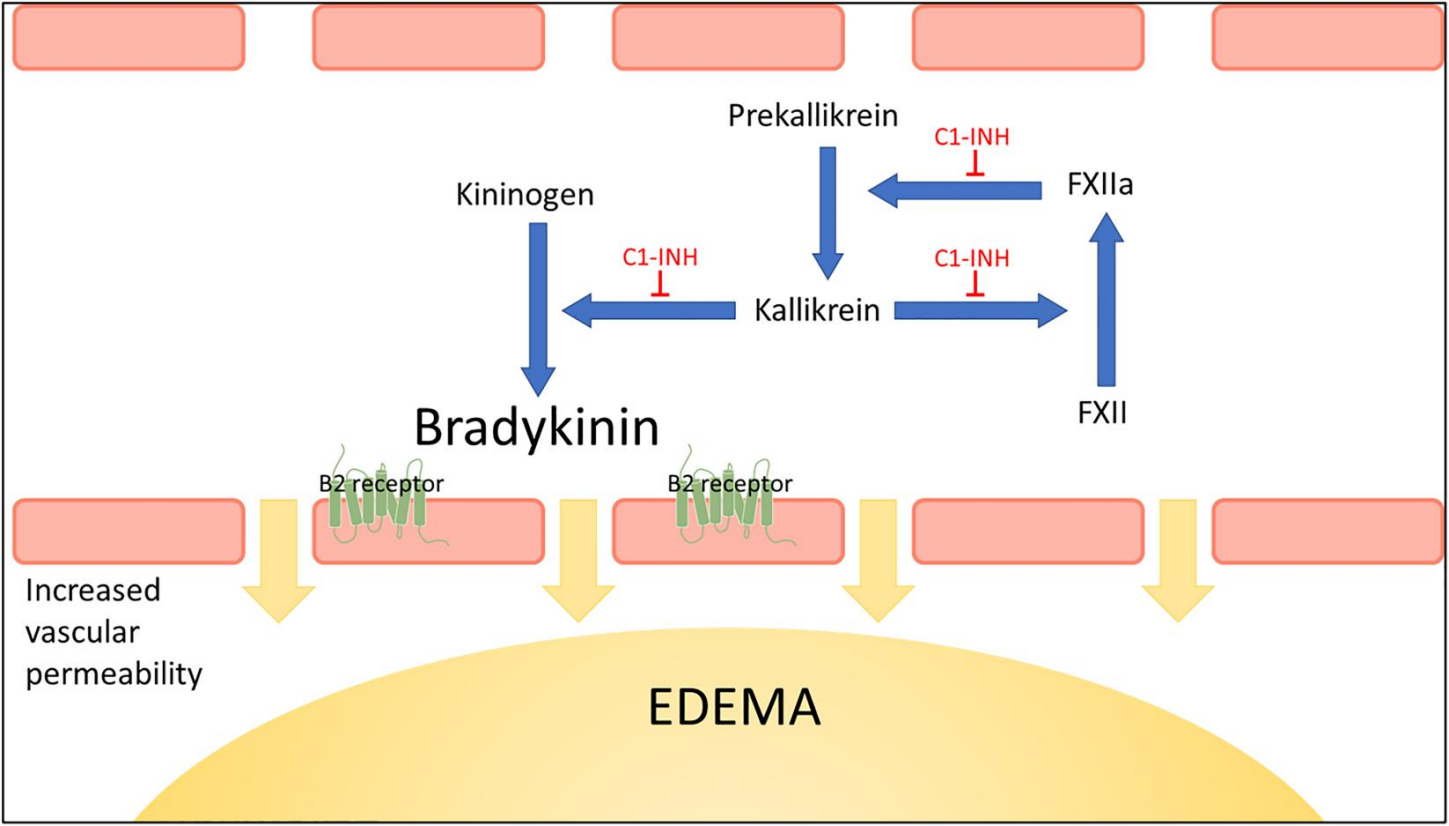
Regulation of the kinin‐forming cascade by C1‐INH. In HAE type I and HAE type II, lack of C1‐INH leads to uninhibited activation of the kallikrein‐kinin system: FXIIa activates prekallikrein to liberate plasma kallikrein, which cleaves kininogen to release bradykinin. FXIIa and kallikrein activate each other in a positive feedback loop resulting in bradykinin overproduction with consequent increased vascular permeability leading to angioedema. C1‐INH regulates these steps to prevent overproduction of bradykinin and subsequent edema.^82^ C1‐INH, C1‐esterase inhibitor; FXII, factor 12; HAE, hereditary angioedema.

Hereditary angioedema (HAE) is a rare autosomal dominant disease characterized by submucosal and skin swelling. It is caused by a deficiency in serum protein levels (type I HAE) or limited functionality (type II HAE) of the plasma protein C1‐INH, leading to bradykinin overproduction and consequent increased vascular permeability, resulting in angioedema (Figure 1).^1^, ^2^

The prevalence of HAE has been estimated at around 1 in 50,000 of the population.^3^ Although the majority of patients have the autosomal dominant inherited form of the disease, 20%–25% develop the condition as a result of spontaneous mutations; 80%–85% of HAE cases are type I and 15%–20% are type II.^4^ Some patients are diagnosed with HAE with normal C1‐INH level and function (HAE‐nC1‐INH), for which causative mutations in six genes have been identified to date.^4^, ^5^

Although rare in infants, clinical symptoms of HAE usually manifest in the first or second decade of life^4^ with a median age of first edema of 11 years.^6^ Owing to its rarity, HAE is often poorly recognized, leading to misdiagnoses or diagnostic delay and, consequently, to inadequate or unnecessary treatment and surgical procedures,^7^, ^8^, ^9^ resulting in a high morbidity and mortality risk for patients.^10^ Diagnosis is often delayed by years, partly because signs and symptoms are similar to other, more prevalent diseases. Low awareness of HAE among healthcare professionals (HCPs) and the multifaceted nature of the condition also contribute to diagnostic delay.^11^, ^12^

Hereditary angioedema is clinically characterized by recurrent severe cutaneous swelling of the face, extremities, and genitals. The mucosal lining of internal organs, mainly the gastrointestinal (GI) and upper respiratory tracts, can also be affected. Depending on the body site involved, HAE can lead to disfigurement, dysfunction, and severe pain, and severely impacts quality of life (QoL).^13^ Furthermore, laryngeal swelling can result in airway obstruction and asphyxiation,^10^ underscoring the importance of early diagnosis. In a retrospective study of 209 patients with C1‐INH deficiency‐related HAE, recurrent cutaneous swelling, GI attacks and laryngeal edema were reported by 96.2%, 93.3% and 52% of patients, respectively.^2^ Nevertheless, misdiagnosis remains a challenge to optimal HAE management. A survey conducted in Germany found that approximately 50% of participant patients with the condition had been misdiagnosed at least once (Table 1).^1^ The most common misdiagnoses included appendicitis, allergy, mental disorders, tonsillitis, and ‘nervous stomach’. The median duration of diagnostic delay, from the first appearance of symptoms to the date of the final diagnosis, was approximately 15 years.^1^

**TABLE 1:** Misdiagnoses (≥1) in patients with HAE.

	*N* = 81	
	**n**	%
Patients reporting misdiagnosis		
Yes	40	49.4
No	41	50.6
Most common misdiagnoses		
Appendicitis	16	40.0
Allergy	12	30.0
Mental disorder	6	15.0
Tonsillitis	3	7.5
‘Nervous stomach’	3	7.5

*Note*: Adapted from Magerl *et al*., 2020^1^. http://creativecommons.org/licenses/by/4.0/.

Abbreviation: HAE, hereditary angioedema.

Current strategies for the treatment of HAE include C1‐INH replacement therapy,^14^, ^15^ bradykinin inhibition with B_2_ receptor antagonists,^14^, ^15^ and therapies targeting plasma kallikrein.^15^ Although effective treatments are available for both on‐demand treatment of attacks and prophylaxis,^15^ the high level of heterogeneity of HAE poses significant challenges to the management of the condition. Increased awareness among HCPs of the multifaceted nature of HAE and comprehensive integrated care that promotes collaboration across clinical settings are paramount to making early, accurate diagnoses and optimizing treatment outcomes.^16^

The recent WAO/EAACI guideline recommends that patients are treated by specialists with expertise in managing HAE; however, it is acknowledged that there are currently not enough HAE‐specialist physicians or angioedema centers for this to always be possible.^17^ Medical facilities providing acute care, including Emergency Departments, are also advised to educate staff in recognizing and treating abdominal and laryngeal attacks, and to design and implement processes to manage HAE.^17^ This review aims to highlight the need for disease awareness and collaboration across different medical specialties in order to appropriately diagnose and treat patients with HAE, while providing specific considerations for the relevant medical specialties. Patient cases are included to illustrate how a patient might present in a particular medical setting.

## 2 HAE IN THE EMERGENCY ROOM

*Patient case*

*A 24‐year‐old female presented at the ER with extreme abdominal pain and vomiting, which started 2 h earlier. She had no other relevant diseases or known allergies and was not taking any medication. The patient was treated with anti‐emetics, analgesics, and fluid replacement therapy, but her symptoms persisted. For that reason, an abdominal imaging test was performed. Her abdominal scan revealed edema of the bowel, free fluid in the abdomen, and mild ascites. Results from tests conducted up to this point indicated angioedema (decreased C4 levels: 30 mg/L). She confirmed that she had no family history of swellings or abdominal pain, although she had abdominal symptoms in the past that required intensive care for hypovolemic shock due to “gastroenteritis”. Following triage with a dedicated rapid tool,* ^18^ *and due to a lack of response to antiallergic therapies (corticosteroids, antihistamines and epinephrine), the history of episodes of abdominal pain, and decreased C4 levels, HAE was suspected, for which she received intravenous (IV) plasma‐derived C1 inhibitor (plasma‐derived C1‐INH). Her abdominal attack resolved over the course of 2 h*.

Diagnosing HAE in the ER is a challenge, even more so when the attacks are predominantly abdominal. The symptoms are misdiagnosed as more common conditions such as appendicitis, pancreatitis, and gastroenteritis. In addition, in cases where patients present peripheral attacks, the symptoms are often misdiagnosed as allergic angioedema; indeed, most angioedema cases in the ER are mast cell‐mediated.^19^ Understanding the clinical differences between mast cell‐ and bradykinin‐related angioedema is therefore crucial in the ER. Of particular importance are features such as family history, early onset of symptoms, slow progression of symptoms during an attack, absence of wheals, no hypotension, lack of response to prior anti‐allergic therapies, and GI or respiratory tract involvement (Table 2).^5^, ^20^, ^21^

**TABLE 2:** Clinical and therapeutic differences between histaminergic and non‐histaminergic angioedema.^5^, ^20^, ^21^

Clinical feature	Histamine‐mediated angioedema	Bradykinin‐mediated AAE	Bradykinin‐mediated HAE
Age of first symptoms	Any	4^th^ – 6^th^ decade	1st–2nd decade
Onset	Rapid (minutes)	Slow (hours)	Slow (hours)
Duration	12–14 h	48–72 h	48–72 h
Family history	No	No	Yes
Cutaneous involvement (other than angioedema)	Wheals present/history of recurrent wheals	Erythema marginatum very rarely	Erythema marginatum prior to an attack is frequent
Predominant location	Face (eyelids/lips)	Face, peripheral, upper airways, GI tract	Face, peripheral, upper airways, GI tract
Past recurrent abdominal pain/swelling	No	Yes	Yes
Anti‐allergy therapies	Effective	Not effective	Not effective

Abbreviations: AAE, acquired angioedema; GI, gastrointestinal; HAE, hereditary angioedema.

The HAE Rapid Triage (HAE‐RT) tool has recently been developed from a Delphi consensus survey to facilitate the diagnosis of HAE in ER settings.^18^ The steps in the HAE‐RT prototype for patients presenting to the ER with recurrent angioedema are as follows: Step 1. Assess and secure airway stability; Step 2. Consider HAE if the patient does not respond to allergy treatments (i.e., antihistamines, corticosteroids and/or epinephrine) and the patient has a history of recurrent abdominal pain or swelling; Step 3. Promptly treat with plasma‐derived C1‐INH, icatibant (a bradykinin B2 receptor antagonist; Table 3), or fresh‐frozen plasma (if others are not available); Step 4. Refer to an allergy specialist to confirm diagnosis. The tool was validated in a retrospective analysis of 107 patient charts (66 with HAE, 41 non‐HAE). Of the predictor variables included in the Delphi questionnaire, four (recurrent episodes of angioedema, past recurrent abdominal pain/swelling, no response to allergy therapy, family history of HAE) were associated with high sensitivity and specificity for identifying patients with HAE, with the latter three factors being sufficient to differentiate between HAE and non‐HAE conditions.^18^ Formal diagnosis of HAE is made following blood tests for C1‐INH concentration and function, and C4.^22^ Due to the length of time required to perform tests for C1‐INH concentration, diagnosis is not feasible in an emergency setting; thus, there is currently an unmet need for the development of biomarkers for rapid, point‐of‐care testing to confirm the suspicion of HAE in the ER. However, C4 levels, which are also typically low in HAE type I and II, can aid the diagnosis of HAE in the ER and help to differentiate HAE attacks from other causes of swelling.^17^, ^22^

**TABLE 3:** Recommended first‐line treatments for patients with HAE.^17^

Indication	First‐line adolescents ≥12 years/adults	First‐line pediatrics (<12 years)
Acute/on demand	pdC1‐INH (IV)	pdC1‐INH (IV)
	rhC1‐INH (IV)	rhC1‐INH (IV)^a^
	Ecallantide (SC)	Icatibant (SC)^a^
	Icatibant (SC)	
Short‐term prophylaxis	pdC1‐INH (IV)	pdC1‐INH (IV)^a^
Long‐term prophylaxis	pdC1‐INH (IV/SC)	pdC1‐INH (IV)^b^
	Lanadelumab	Tranexamic acid^c^
	Berotralstat	

*Note*: Availability and indications of treatments vary between countries.

Abbreviations: HAE, hereditary angioedema; IV, intravenous; pdC1‐INH, plasma‐derived C1‐esterase inhibitor; rhC1‐INH, recombinant human C1‐esterase inhibitor; SC, subcutaneous.

^a^ ≥2 years.

^b^ ≥6 years.

^c^ When C1‐INH is not available.

Diagnosed patients with HAE may require ER care owing to the lack of access to home treatment, unavailable caregivers at home, continuing or returning symptoms despite on‐demand home therapy, or as part of their treatment plan. Hereditary angioedema guidelines recommend that all patients with HAE should have a treatment action plan, including an emergency plan, and that the local hospital should have HAE‐specific therapies available for the emergency treatment of attacks.^16^, ^17^, ^22^ Indeed, having a treatment plan in place has been associated with a greater likelihood of receiving HAE‐specific therapy in the ER, compared with not having one (99% vs. 74%).^23^ An example action plan for on‐demand treatment has recently been published, which should clearly outline which medications to use to treat attacks, and how to administer them.^3^

Hereditary angioedema management in the ER setting needs to be improved through increased awareness, new rapid diagnostic tools, and availability of effective, HAE‐specific therapies.^23^

**Key points for emergency medicine**

- Understanding the clinical differentiation between mast cell‐mediated and bradykinin‐mediated angioedema is crucial for correct treatment and rapid alleviation of symptoms in the ER
- Strong indicators that a patient has HAE are as follows:
  - Past recurrent abdominal pain/swelling
  - Family history of HAE
  - No response to antiallergic therapies
- All patients with a diagnosis of HAE should have an emergency action plan, with access to HAE‐specific therapy at their local hospital

## 3 HAE AND PEDIATRICS

*Patient case*

*A 7‐year‐old girl presented to the clinic with intense abdominal pain, vomiting and diarrhea, and a swollen right hand. The symptoms started 12 h before presentation and were preceded by a non‐pruritic skin rash. There was no family history of HAE, swelling episodes, or unclear death. The patient was administered oral antihistamines and cortisone, with no response, and subsequently underwent abdominal ultrasound that revealed GI swelling. Due to the typical garland‐like skin rash typical of the HAE prodrome, erythema marginatum, and lack of response to oral antihistamines and cortisone, she was administered IV plasma‐derived C1‐INH at a dose of 20 IU/kg over 5–10 min and her symptoms resolved within 2 h. Further blood tests demonstrated low C1‐INH concentration (100 mg/L, normal range 210–380 mg/L), low C1‐inhibitor function (32%, normal >70%) and low C4 levels (80 mg/L, normal range 100–400 mg/L), confirming type I HAE*.

As the signs and symptoms of HAE usually develop in childhood or adolescence,^17^ diagnosing the condition at an early age is vital, and pediatricians are ideally positioned for this. Although in girls the first symptoms of HAE usually develop in puberty, with hormonal changes acting as a trigger for HAE attacks,^24^ in all pediatric patients, symptoms tend to increase in frequency with increasing age.^17^

**FIGURE 2:**
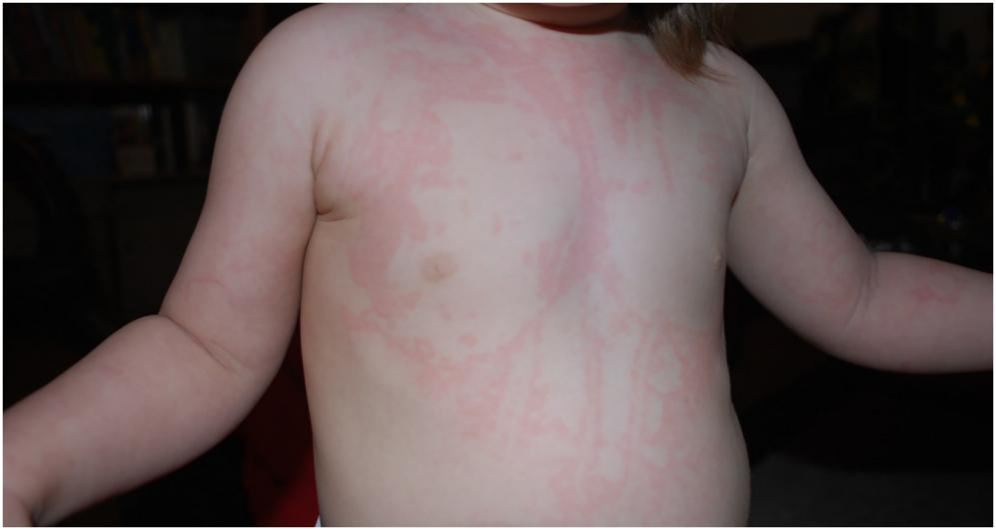
Erythema marginatum – a prodromal cutaneous signal of an HAE attack. Image from personal collection (M Magerl) and used with consent. HAE, hereditary angioedema.

Erythema marginatum is a useful prodromal sign with high prevalence, affecting 42%–58% of pediatric patients with the condition, in addition to some adult patients.^17^, ^25^ It may be the initial symptom of C1‐INH HAE and can manifest without subsequent edemas, particularly in children.^26^ Erythema marginatum is a flat, non‐pruritic, ring‐shaped rash that most commonly affects the trunk (Figure 2)^25^ and can manifest minutes to days before the development of swelling,^26^ thereby facilitating early recognition and prevention.^25^

Comprehensive integrated care across healthcare settings, with input from family and teachers, is crucial to achieving effective disease management.^27^ Screening of children from HAE‐affected families is recommended in order to diagnose the condition before the onset of symptoms.^17^ Measuring C1‐INH levels, C4 levels, or carrying out genetic testing can achieve this, though screening options may vary between countries. Treatment options for pediatric patients with HAE include on‐demand therapy with plasma‐derived C1‐INH, recombinant C1‐INH, and icatibant, as well as pre‐procedural (e.g., before dental extractions) and long‐term prophylaxis with plasma‐derived C1‐INH (Table 3). More recently, additional long‐term prophylaxis options have been made available for adolescent patients; a subcutaneous formulation of plasma‐derived C1‐INH, and the plasma kallikrein inhibitors lanadelumab (subcutaneous injection) and berotralstat (oral), are now approved for prophylactic use in patients ≥12 years old.^28^, ^29^ Attenuated androgens are generally not recommended for use in children and adolescents prior to Tanner Stage V, owing to a substantial risk for adverse events (AEs) such as interference with natural growth and maturation.^17^, ^30^ Although tranexamic acid may be used for short‐term or perioperative prophylaxis in children, it is believed to be ineffective in some pediatric patients and that plasma‐derived C1‐INH is a more appropriate choice.^27^

Of note for adolescents, estrogen and estrogen‐containing oral contraceptives may trigger HAE attacks in females.^17^, ^31^ A link between estrogen and pathways associated with HAE is well established.^32^, ^33^, ^34^, ^35^ Estrogen is associated with increased Factor XII, kallikrein, and kinin concentrations and has a role in the regulation of B2 receptor gene expression and function.^34^, ^36^, ^37^ The result of these alterations is C1‐INH consumption,^38^, ^39^ and as such, decreased plasma levels of C1‐INH in healthy women taking oral contraceptives have been observed.^37^ Accordingly, estrogen exposure is associated with worsening of the frequency and severity of HAE symptoms.^34^

In the FXII‐HAE form of HAE with normal C1‐INH, symptoms may show pronounced estrogen dependency, worsening under hyper‐estrogenic conditions, such as pregnancy, oral contraceptive use, and in vitro fertilization. By contrast, progestin oral contraceptives have not been reported to precipitate attacks, and have in fact been found to improve HAE symptoms in 82% of female patients, according to a French retrospective study.^31^ Other potential triggers include surgery (e.g., dental extractions), GI or upper respiratory infections, emotional stress, physical over‐exertion, and trauma.^17^ It is important that children, their parents, teachers, and physicians are aware of factors that tend to precipitate attacks.

**Key points for pediatricians**

- Symptoms of HAE often present in childhood and can increase, decrease, or stay the same with age
- Erythema marginatum is a distinctive prodromal symptom that is common in HAE and can occur without subsequent edema
- Effective treatments are available for children and adolescents including long‐term prophylaxis options now available for children and adolescents ≥12 years old; androgens are not recommended due to a high risk of adverse effects

## 4 HAE AND OTOLARYNGOLOGY

*Patient case*

*A 35‐year‐old male patient with no family history of HAE presented to the otolaryngology clinic with recurrent swelling of the eyelids (usually lasting 2–3 days) and, occasionally, of lips and cheeks. He reported that he has never experienced laryngeal swelling. He sometimes experienced a non‐pruritic skin rash and had no known allergies, although lactose intolerance had been suspected in the past due to recurrent abdominal bloating. After being shown photographs of angioedema, he noted that his foot sometimes swelled after hiking and the hands swelled after handcrafting. When asked if he had undergone any abdominal surgery, he confirmed that he underwent an appendectomy at the age of 20 and a laparotomy for adhesive ileus six years later; however, his abdominal symptoms persisted, which he attributed to the lactose intolerance. Reported acute treatment attempts with corticosteroids and antihistamines in the past did not resolve the swellings. Subsequent blood tests showed low concentration and low function of C1‐INH, indicating type I HAE*.

Although otolaryngologists are often required to treat laryngeal angioedema, there is evidence to suggest that they may be inadequately prepared. A survey of program directors (*n* = 34) of otolaryngology residencies in the US found that <50% felt their training program provided adequate education and exposure to HAE, even though 97% thought such knowledge was important for their residents to have.^40^ Awareness of HAE by otolaryngologists is vital, considering the risk of suffocation due to laryngeal edema, which can be fatal if symptoms are not alleviated or airway patency is not quickly restored. As such, the mortality rate of undiagnosed patients with HAE during a laryngeal attack is high (29.4%).^10^ It is important to note that while the frequency of laryngeal attacks is low, around 50% of all patients with HAE experience at least one laryngeal attack in their lifetime, with one study of 123 patients reporting a mean age at first attack of 26.2 years; 80% occurring between the ages of 11 and 45 years.^41^ Early diagnosis, and therefore earlier control of the condition, could prevent a patient from ever experiencing a laryngeal attack, and greatly reduce the mortality risk.^10^ Patients often experience anxiety and distress around laryngeal attacks due to the fear of not being able to breathe or get to emergency care in time.^42^ While uncommon, it is possible that laryngeal edema is the first symptom of HAE experienced by a patient,^43^ again emphasizing the need for otolaryngologists to have a high degree of awareness of HAE.

Factors contributing to the effective management of patients with potential laryngeal edema in the otolaryngology setting include thorough history‐taking to identify non‐obvious symptoms and potential triggers of the attack. For example, a patient may be taking an angiotensin converting enzyme inhibitor (ACEi), such as ramipril, mediator for bradykinin‐mediated angioedema, and trigger for HAE attacks, as a result of bradykinin accumulation.^1^, ^2^, ^44^ Patient input also plays a crucial role in reaching an accurate diagnosis of HAE. The authors' experience has shown that photographs may be used to enable patients to differentiate between HAE and non‐HAE clinical features (e.g., allergy‐related wheals vs. angioedema or mast cell‐mediated rash vs. erythema marginatum), thereby facilitating accurate reporting of symptoms.

Importantly, manipulation of the mouth and throat should be avoided as this may exacerbate the attack. Transnasal endoscopy can be used to assess laryngeal status as the nasal mucosa is not susceptible to bradykinin‐induced angioedema, for reasons unknown. If symptoms progress, the airways should be secured by protective intubation, preferably transnasal, if the tongue is swollen. Coniotomy (*syn*. cricothyrotomy) should be performed expeditiously if intubation is not successful.^41^

Awareness of appropriate treatment is as important as early diagnosis to improve patient outcomes. While mast cell‐mediated angioedema responds to antihistamines and corticosteroids, this is not the case for bradykinin‐mediated angioedema, such as in HAE or ACEi‐induced angioedema, which do not respond to this approach. If HAE is suspected, HAE‐specific treatment such as C1‐INH or bradykinin receptor antagonist should be administered as soon as possible, and the patient should be referred to an HAE center for formal diagnosis and treatment optimization. Currently, there is no licensed therapy for ACEi‐induced angioedema, but the drug should be discontinued, and the airways secured if needed. The emotional state of the patient should also be considered, as laryngeal attacks are a frightening experience associated with high levels of anxiety.^13^, ^42^

**Key points for otolaryngologists**

- Although rare, laryngeal edema can be the first symptom of an undiagnosed patient with HAE and can be fatal if not managed appropriately
- Obtaining a thorough patient history and symptoms outside of otolaryngology is vital in the differential diagnosis of HAE
- In patients undergoing a suspected laryngeal attack of HAE, avoid manipulation of the mouth and tongue, use transnasal endoscopy to assess the larynx, and perform expeditious transnasal intubation or coniotomy (*syn*. cricothyrotomy) to secure airway patency

## 5 HAE AND GASTROENTEROLOGY

*Patient case*

*A 33‐year‐old female patient presented at the Gastroenterology Unit to undergo laboratory tests for suspected food allergy. She was hospitalized for the first time at the age of 9 years with GI symptoms (abdominal pain, distention, diarrhea), occasionally associated with skin edema (which frequently presented in the absence of pruritus or urticaria), as well as laryngeal edema, dysphonia, and dyspnea. Her episodes occurred about twice a month and were unresponsive to antihistamines and corticosteroids. Since the symptoms often presented after the consumption of some stone fruits and nuts (i.e., peaches, hazelnuts, peanuts, and almonds), the patient initially underwent a Skin Prick Test and a specific IgE assessment for culprit foods, which showed positive results (Specific IgE for peach: 15.2 KU/L, peanuts: 3.84 KU/L, hazelnut: 7.94 KU/L, almond: 1.6 KU/L; reference range: <0.35 KU/L). The patient was consequently diagnosed with food allergy and started an elimination diet. The patient's 6‐year‐old daughter also complained of recurrent abdominal pain, which often presented after the consumption of peaches, apples, hazelnuts, and peanuts. An allergological workup confirmed the suspicion of food allergy; therefore, the girl also started an elimination diet. Despite good adherence to the elimination diet, both patients continued to experience cyclical GI symptoms sometimes associated with non‐pruritic skin edema. In addition, an abdominal ultrasound performed during an episode of acute abdominal pain revealed transient bowel edema and ascites. Given the family history, the persistence of the attacks despite the elimination diet, and the inefficacy of the on‐demand therapy with antihistamines and corticosteroids, bradykinin‐mediated angioedema was suspected. Both patients underwent relevant laboratory tests, which showed undetectable C1‐INH antigen, reduced C1‐INH activity assay (11% for the patient and 30% for her daughter; normal values: ≥50%), and decreased C4 levels (30 mg/L for the patient and 40 mg/L for her daughter; normal values 100–400 mg/L). The genetic tests showed that the two patients shared the same mutation in* *SERPING1*, *confirming the diagnosis of HAE type I. The patients were then effectively managed with C1‐INH replacement therapy (i.e., plasma‐derived C1‐INH at a dose of 20IU/kg) to treat attacks with good clinical response*.

This patient case is typical of HAE, as GI symptoms are common in this condition, affecting 90% of patients. Of all patients presenting to ER, a large proportion (7%–10%) are due to acute abdominal pain,^45^, ^46^, ^47^ and 29% of all HAE cases present with GI symptoms only, making the diagnosis of HAE extremely challenging.^48^ Common GI manifestations of HAE include nausea and vomiting, pain, abdominal distension, ascites and diarrhea.^8^ Unusual GI manifestations may include hypovolemic shock, retroperitoneal angioedema, high transaminase levels, and hepatic parenchymal changes.^49^

Food ingestion has recently been suggested as a trigger for HAE abdominal attacks^50^, ^51^; however, the way in which foods can trigger HAE attacks is currently unknown. Some authors have hypothesized that IgE‐mediated hypersensitivity with mast cell activation may promote bradykinin generation through the release of heparin,^46^, ^47^ while others believe that the induction of attacks is more likely associated with a histamine intolerance reaction.^51^, ^52^ In addition, food allergy may resemble HAE attacks, since HAE clinical features may mimic those of anaphylactic shock, thus complicating the differential diagnosis with consequent diagnostic delay.

The severity of HAE can be aggravated by other concomitant GI disorders (e.g., food allergy, celiac disease, Crohn's disease, or *Helicobacter pylori* infection).^49^ For example, the presence of *H*. *pylori* has been associated with significantly higher frequency of acute abdominal pain in HAE, triggered by humoral immune response activation, leading to C1‐INH depletion.^53^ Furthermore, direct local mucosal damage due to cell death may lead to the activation of complement and other contact systems.^53^ Screening patients with HAE for *H*. *pylori* is warranted as successful eradication has been shown to significantly reduce the number of attacks.^53^

Owing to its rarity and heterogeneity, HAE is frequently misdiagnosed as its GI manifestations are attributed to other conditions, such as appendicitis, cholecystitis, pancreatitis, celiac disease, food allergy, and inflammatory bowel disease, often leading to unnecessary surgical procedures.^49^ It has been estimated that patients with HAE are 2.5 times more likely than those without the condition to undergo abdominal surgery.^9^ A web‐based survey of 107 Chinese patients found that 24.7% underwent an unnecessary appendectomy or laparotomy.^7^

An important consideration, when managing patients with suspected HAE‐related GI symptoms, is that the instrumentation of the oropharynx should be avoided, as it can trigger a life‐threatening laryngeal attack.^4^, ^54^ Imaging techniques may help rule out other causes of abdominal symptoms and may reveal edema of the gut and ascites often present during abdominal attacks of HAE.^54^

**Key points for gastroenterologists**

- 90 percent of patients with HAE experience abdominal symptoms, and around a third of patients experience solely GI symptoms
- Correct diagnosis following abdominal attacks is challenging as symptoms are similar to more common conditions, resulting in high rates of unnecessary surgery
- Some GI disorders, such as *H*.*pylori* infection, may aggravate HAE symptoms
- Instrumentation of the oropharynx should be avoided in patients with suspected HAE as this may trigger a laryngeal attack

## 6 HAE AND DERMATOLOGY

*Patient case*

*A 28‐year‐old male patient reported recurrent swellings mainly of the extremities, less frequently of the face and the genitals, which had been occurring since he was about 14 years old. At first, the complaints occurred every three to 4 months, but since he started working 4 years ago, the complaints have occurred every six to 8 weeks. The patient himself establishes a connection between stress and the occurrence of the attacks. In addition, he reported that the attacks are almost always accompanied by the appearance of a reddish, non‐elevated and non‐itching skin rash on the trunk. The skin manifestations appear before the actual swelling and are described by the patient as a “giraffe‐like” reticular pattern. In the early days of his history, medical attention was sought for each attack, usually at the emergency department or by the pediatrician on duty. As a rule, treatment consisted of anti‐allergic therapy followed by monitoring. A relatively thorough allergological diagnosis was made in which sensitization against early blossom and grass pollen was proven. In later years, the patient sought medical help only in the case of more severe complaints because, in his experience, medical interventions were hardly successful in the past. Since the age of 16, extremely painful attacks would also occur about once or twice a year. Neither the patient nor the treating doctors established a connection between the abdominal complaints and the swelling events, and always considered and treated them separately. During one such painful abdominal attack, he had his appendix surgically removed when he was 17 years old. Besides this surgery, the treatment of the abdominal attacks was always symptomatic. Diagnostics with regard to the abdominal complaints were largely limited to the diagnosis of food intolerance reactions. When taking a history in our dermatology clinic, the patient was surprised when he was asked about recurrent abdominal symptoms and answered affirmatively. The family history was initially given as negative; however, later it was discovered that the father (an only child) also suffered from HAE but had been largely symptom‐free during his life. The patient's grandparents had already passed away and the patient had no siblings. C1‐INH and C4 laboratory tests were ordered, which confirmed the patient's diagnosis of HAE (C1‐INH concentration: 60 mg/L; C1‐INH function: 23%; C4: 80 mg/L; splice site mutation in intron 7). The patient was informed about the nature of the course of the disease and was prescribed icatibant (SC) 30 mg as on‐demand treatment of emerging attacks. Furthermore, he was educated about the warning function of the prodromal sign, erythema marginatum*.

Patients with angioedema that is not life‐threatening, such as that affecting the extremities, may visit a dermatologist for investigation and treatment. Skin swellings are one of the most common manifestation of HAE attacks, accounting for 50% of all HAE attacks and affecting 96% of patients in a German study.^2^ Patients may also present to dermatologists with the prodrome, erythema marginatum. As such, dermatologists are likely to encounter a patient with HAE during their clinical career.^55^ Most cases of recurrent angioedema turn out to be chronic spontaneous urticaria with or without wheals, but bradykinin‐mediated angioedema must always be considered.^5^ The physician must be alert when patients deny the occurrence of itchy wheals but instead report the typical erythema marginatum that often precedes the attacks. In contrast to wheals, these are not itchy, not raised, and the erythematous areas are usually ring‐shaped and not two‐dimensional. The distinction between erythema marginatum and wheals should be easy for the dermatologist, but in practice, erythema marginatum is often misinterpreted as urticaria or at least as an allergic concomitant. This misinterpretation apparently solidifies the suspected diagnosis of allergic angioedema. It has been shown that patients with HAE and the occurrence of the prodromal sign erythema marginatum have to wait 4 years longer for the correct diagnosis of HAE compared to patients who do not show erythema marginatum (11 instead of 7 years).^56^

Medication is a common cause of angioedema without wheals. ACEi‐induced angioedema accounts for 30%–60% of ER visits for angioedema without wheals, usually affecting the face and oral and pharyngeal mucosa with episodes lasting 24–72 h.^57^ The reaction often occurs within the first month of therapy, but may occur years after commencing treatment,^58^ and the reaction is not related to dose.^59^ ACEi‐angioedema has an incidence of 0.23% during the first year of treatment, with significantly higher frequency in Black and Hispanic patients.^60^ Screening with a blood test for C4 levels may help differentiate HAE from ACEi‐induced angioedema; levels are low in HAE types I and II and normal in ACEi‐induced angioedema.^57^ In addition to drug‐induced angioedema, dermatologists must also be aware of the distinction from conditions that may resemble angioedema or ‘pseudoangioedema’. These conditions and their distinguishing clinical properties from angioedema are listed in Table 4.^61^

**TABLE 4:** Differential diagnoses in ‘pseudoangioedema.’^61^

Condition	Differential diagnosis
Subcutaneous emphysema^70^	Crepitus – a crackling sensation as the gas is pushed through the tissue upon palpitation
Acute contact dermatitis^71^	Superficial erythema, dermatitis, pruritis, peeling when swelling resolves, responds to corticosteroids
Hypothyroidism^72^	Non‐transient. Low levels of thyroid hormones
Superior vena cava syndrome^73^	Vein distension across the chest and neck and increased signs when in supine position. Diagnosed by chest scan including thoracic inlet
Dermatomyositis^74^	Accompanying muscle weakness, weight loss, and red‐purple erythematous rash around eyes
Chronic eyelid edema and rosacea (Morbus Morbihan)^75^	Develops gradually over months to years. Restricted to forehead, glabella, upper eyelids, and cheeks
Orofacial granulomatosis^76^	Chronic presentation
Hypocomplementemic urticarial vasculitis syndrome^77^	Urticarial skin lesions, hyperpigmentation or purpura upon resolution
Capillary leak syndrome (Clarkson's disease)^78^	Generalized and symmetrical swelling, hypovolemia, hemoconcentration, and reduced serum albumin
Episodic angioedema with hypereosinophilia (Gleich's syndrome)^79^	Weight gain, fever, pruritus, sometimes urticaria
Cluster headache^80^	Presence of headache, conjunctival injection, ptosis, pupil constriction, watering of eyes
Idiopathic edema^81^	Pitting edema, periorbital edema after recumbency overnight, weight gain from morning to evening

Correct diagnosis and timely intervention by dermatologists are vital in order to prevent future attacks that may warrant emergency care. It is recommended that patients presenting to dermatologists with recurrent swelling episodes without urticaria and no suspected allergy should be promptly tested for HAE (C4 levels, C1‐INH protein levels and C‐INH function).^55^

**Key points for dermatologists**

- Edema of the skin is the most common symptom of HAE; it affects the inner dermis and subcutaneous tissue and is non‐pruritic
- Dermatologists may also encounter the prodromal erythema marginatum rash, characteristic of HAE
- If a patient fails to respond to antihistamines/corticosteroids, it is important for dermatologists to consider bradykinin‐mediated angioedema; HAE may be distinguished from ACEi‐mediated angioedema by a blood test for C4 levels

## 7 LOOKING FORWARD

The HAE treatment landscape is changing rapidly, as new therapies recently made available and others currently under development promise to improve the management of the disease, while providing patients with more convenient administration and greater treatment flexibility.

Emerging therapies for long‐term prophylaxis of angioedema attacks include antisense inhibitors of prekallikrein and drugs targeting excess FXII activation.^62^, ^63^ Gene therapy is also being investigated, as this could prevent acute episodes, while reducing the burden of repeated medication use; however, its safety and tolerability in HAE is currently unknown.^62^ Additionally, there are a number of emerging investigational oral therapies that aim to treat attacks, such as a plasma kallikrein inhibitor^64^ and bradykinin B2‐receptor antagonist.^65^

Access to therapies and expert centers for patients with HAE remains limited in some countries. However, progress is being made through the work of the Angioedema Centers of Reference and Excellence (ACARE) network (https://acare‐network.com/), which aims to promote awareness of angioedema and facilitate patient access to HAE therapies, both in countries where they are available and in those where they are not.

A shift in treatment paradigm, from acute treatment to long‐term prophylaxis, is encouraged and expected. By preventing acute episodes of HAE, prophylaxis can help patients avoid needless medication and surgical procedures. It can also reduce the number of hospitalizations and time off work/school, in addition to providing health and QoL benefits for patients.^66^, ^67^ One challenge in the future may be ensuring that patients remain vigilant for attacks and do not become complacent and stop taking treatment due to an extended attack‐free period.

Reductions in diagnostic delays are also expected, and urgently needed. European data from the Icatibant Outcome Survey registry revealed a median diagnostic delay of 8.5 years (range 0–62 years).^12^ Similarly, in a US survey of patients with HAE, although 33% of respondents reported they had been diagnosed within a year of their first symptoms, 32% reported a diagnostic delay of ≥10 years.^68^ Recent data, however, do indicate that there has been some improvement in HAE diagnosis over time.^69^ Further improvements are needed, which may be achieved through increasing both health care provider and patient knowledge and awareness of HAE, and by promoting collaboration across clinical settings, health services and countries.

## AUTHOR CONTRIBUTIONS

**Markus Magerl**: Conceptualization (equal); Writing – original draft (equal); Writing – review & editing (equal). **Anna Sala‐Cunill**: Conceptualization (equal); Writing – original draft (equal); Writing – review & editing (equal). **Christina Weber‐Chrysochoou**: Conceptualization (equal); Writing – original draft (equal); Writing – review & editing (equal). **Susanne Trainotti**: Conceptualization (equal); Writing – original draft (equal); Writing – review & editing (equal). **Ilaria Mormile**: Conceptualization (equal); Writing – original draft (equal); Writing – review & editing (equal). **Giuseppe Spadaro**: Conceptualization (equal); Writing – original draft (equal); Writing – review & editing (equal).

## CONFLICT OF INTEREST STATEMENT

Medical writing assistance was provided by Meridian HealthComms Ltd., funded by CSL Behring. MM has received grants/contracts, consulting fees, and honoraria for presentations from CSL Behring, Takeda, Kalvista, New Bridge Pharma, Pharvaris, BioCryst and Astria; support for attending meetings and/or travel from Takeda, New Bridge Pharma and Kalvista; has participated on a Data Safety Monitoring Board/Advisory Board for Kalvista and Pharvaris; and has received equipment/materials/drugs/medical writing/gifts/other services from Takeda. AS‐C has received honoraria for presentations from CSL Behring, Takeda, Novartis, Sanofi and Pfizer; payment for expert testimony and support for attending meetings and/or travel from CSL Behring, Novartis and Sanofi; and has participated on a Data Safety Monitoring Board/Advisory Board for Takeda, Novartis, Sanofi and Pfizer. CW‐C has received support for attending meetings and/or travel from Takeda. ST has received honoraria for lectures from CSL Behring, Takeda and RG Gesellschaft für Information und Organisation mbH; payment for expert testimony and support for attending meetings and/or travel from CSL Behring and Takeda; and has participated on an Advisory Board for Takeda. IM and GS report no conflict of interest.

## Data Availability

Data sharing not applicable to this article as no datasets were generated or analyzed during the current study.
